# *β*-Catenin and NF-*κ*B co-activation triggered by TLR3 stimulation facilitates stem cell-like phenotypes in breast cancer

**DOI:** 10.1038/cdd.2014.145

**Published:** 2014-09-26

**Authors:** D Jia, W Yang, L Li, H Liu, Y Tan, S Ooi, L Chi, L G Filion, D Figeys, L Wang

**Affiliations:** 1 Department of Biochemistry, Microbiology and Immunology, Faculty of Medicine, University of Ottawa, 451 Smyth Road, Ottawa, Ontario, Canada K1H8M5; 2 Life Science College of Northwest A&F University, Yangling, Shaanxi 712100, China; 3 Key Laboratory for the Genetics of Developmental and Neuropsychiatric Disorders, Bio-X Institutes, Ministry of Education, Shanghai Jiao Tong University, Shanghai 200240, China; 4 Department of Neurosurgery, Qilu Hospital of Shandong University, Jinan, Shandong 250012, China; 5 Department of Chemistry, University of Ottawa, Ottawa, Ontario, Canada; 6 Regenerative Medicine Program, Ottawa Hospital Research Institute, Ottawa, Ontario, Canada; 7 Ottawa Institute of Systems Biology, University of Ottawa, Ottawa, Ontario, Canada

## Abstract

Cancer stem cells (CSCs) are responsible for tumor initiation and progression. Toll-like receptors (TLRs) are highly expressed in cancer cells and associated with poor prognosis. However, a linkage between CSCs and TLRs is unclear, and potential intervention strategies to prevent TLR stimulation-induced CSC formation and underlying mechanisms are lacking. Here, we demonstrate that stimulation of toll-like receptor 3 (TLR3) promotes breast cancer cells toward a CSC phenotype *in vitro* and *in vivo*. Importantly, conventional NF-*κ*B signaling pathway is not exclusively responsible for TLR3 activation-enriched CSCs. Intriguingly, simultaneous activation of both *β*-catenin and NF-*κ*B signaling pathways, but neither alone, is required for the enhanced CSC phenotypes. We have further identified a small molecule cardamonin that can concurrently inhibit *β*-catenin and NF-*κ*B signals. Cardamonin is capable of effectively abolishing TLR3 activation-enhanced CSC phenotypes *in vitro* and successfully controlling TLR3 stimulation-induced tumor growth in human breast cancer xenografts. These findings may provide a foundation for developing new strategies to prevent the induction of CSCs during cancer therapies.

Despite incessant efforts to combat cancer over decades, breast cancer is still the second leading cause of death in women, remaining high with over 39 000 deaths in 2012 in the United States alone.^1^ Conventional interventions, such as radiation or chemotherapy, may eliminate the bulk of the tumor but spare rare aggressive cancer cells that have an exceptional capacity to survive, self-renew, and advance the malignancy. These residual tumor cells have recently been found to possess key stem-like properties and have thus been termed ‘cancer stem cells (CSCs)'.^2, 3, 4, 5^ Breast CSCs, characterized by expression of CD44^high^/CD24^−/low^ surface markers, are proposed to be largely responsible for cancer progression and metastasis.^3,6,7^ These CD44^high^/CD24^−/low^ cells possess stem cell-like properties and tumor-initiating capacity. Furthermore, these cells resist standard therapies^3,6,8,9^ and can be converted from non-CSC cells under certain conditions.^10,11^ Therefore, specific targeting of CSCs within a tumor will be imperative to prevent disease progression and recurrence.^5^ However, the conditions and mechanisms underlying CSC formation remain poorly understood. Although the majority of cancers arise from *de novo* oncogenic and epigenetic alterations, most tumors display signals of unremitting inflammatory activity,^12^ which occurs even in the absence of infection or autoimmunity.^13^

Toll-like receptors (TLRs) are a key family of microbial sensors in the host innate and adaptive immunity as well as in tissue repair and regeneration. They are also involved in the inflammatory signaling triggered by endogenous macromolecules released by injured tissue.^14,15^ Ten TLRs are encoded by the human genome. TLRs detecting nucleic acids (TLR3, TLR7, TLR8, and TLR9) are localized in the endosomal compartment in nearly all cell types, while TLRs mainly detecting proteolipidic structures (TLR1, TLR2, TLR4, TLR5, TLR6, and TLR10) are exposed on the cell surface.^14,16^ In cancer, TLRs have emerged as important participants in tumorigenesis. TLR3, 4, 7, and 9 were overexpressed in 70, 72, 67, and 78% of patients with esophageal cancer.^17^ The -196 to -174del/del genotype of TLR2 may increase the risk of gastric cancer,^18^ and TLR4+896A>G polymorphism is a risk factor for non-cardia gastric carcinoma.^19^ Functions of epithelial-expressed TLR2 and 5 in promoting epithelial cell survival, proliferation, migration,^20^ and angiogenesis (TLR2 only)^21^ may be usurped by tumor cells to facilitate progression and metastasis. Although TLR3, 5, 7, 8, and 9 may achieve antitumor effects by converting immune tolerance into antitumor immunity,^14^ considerable discrepancies have been reported. For instance, high TLR3 expression in esophageal cancer cells was significantly associated with a higher probability of lymph-node metastasis and increased depth of invasion.^17^ Elevated TLR3 expression in breast cancer was also associated with poor prognosis.^22,23^

Several clinical trials using TLR agonists for cancer treatment are currently in progress. Among all anticancer immunotherapy agents, TLR agonists are classified as the ones with highest potential. However, clinical outcomes are inconsistent and repeatedly disappointing.^24^ Specifically, high expectations were placed on TLR3 agonists for their ability to boost host immune systems to fight diseases. TLR3 is located in intracellular endosomes for the recognition of double-stranded RNA (dsRNA) and polyinosinic-polycytidylic acid (poly(I:C), a synthetic analog of dsRNA).^25^ In addition to upregulating immune response, a broader range of functions of TLR3 have been revealed recently, especially in stem cells. For instances, activation of TLR3 was found to amplify mesenchymal stem cell trophic factors and enhance therapeutic potency.^26^ Recently, Lee *et al.*^27^ also showed that TLR3 stimulation caused rapid and global changes in the expression of epigenetic modifiers to enhance chromatin remodeling and nuclear reprogramming when converting adult cells to induced pluripotent stem cells. Nevertheless, the role of TLR3 in cancer remains inconsistent, and its function in breast CSCs is unclear.

Here, we demonstrate that TLR3 activation in breast cancer cells leads to a preferential enrichment of a subset of cells with CSC phenotypes *in vitro* and *in vivo*. Conventional NF-*κ*B signaling is not fully responsible for the enhanced CSC properties. Unexpectedly, *β*-catenin pathway is required for the promotion of CSC phenotypes in breast cancer cells following TLR3 activation. Our results provide new tantalizing strategies to effective target breast and other CSCs with elevated TLR3 expression to prevent progression and relapse.

## Results

### TLR3 but not TLR5, 7, and 8 activation associates with breast CSC-like properties

**Figure 1:**
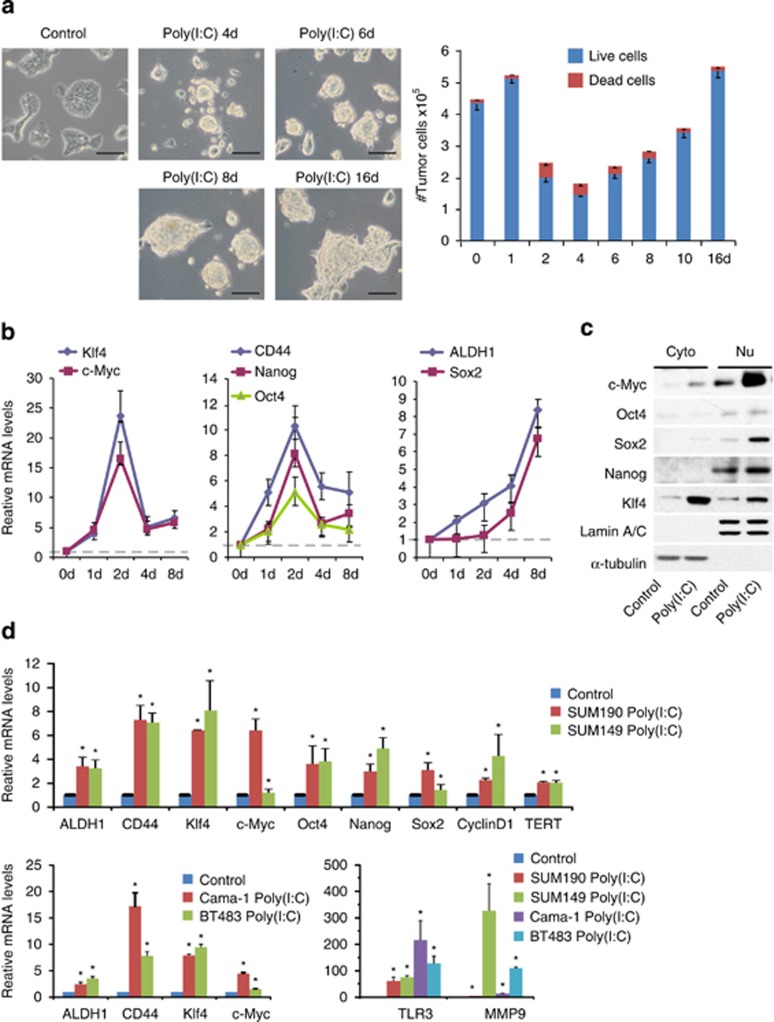
TLR3 but not TLR5, 7, and 8 activation associates with breast CSC-like properties.(**a**) SUM190 cells treated with 1 *μ*g/ml of poly(I:C) for different days show a morphological change with the formation of sphere-like live aggregates. Scale bars, 100 *μ*m. The fractions of viable cells after poly(I:C) treatment for different days were determined by trypan-blue exclusion assay. Bars denote the standard error for proportions (*n*=3). (**b**) Trend changes of stemness genes at days 1, 2, 4, and 8 in poly(I:C)-treated SUM190 cells from three independent experiments. Error bars indicate S.D. (**c**) Western blot analysis of transcriptional factors in SUM190 cells treated with vehicle control or poly(I:C). *α*-tubulin and Lamin A/C: internal loading controls for cytoplasmic (Cyto) and nuclear (Nu) proteins. (**d**) Quantitative real-time PCR analysis of indicated genes in SUM190, SUM149, BT-483, and Cama-1 cells after treatment with 1 *μ*g/ml of poly(I:C) for 4 days. GAPDH mRNA was used to normalize variability in template loading. Data represent the average±S.D., *n*=3; *P<0.05

To determine whether TLR3 stimulation links to CSC phenotypes, we activated TLR3 pathway in inflammatory breast cancer cell line SUM190 using poly(I:C), a specific TLR3 ligand.^25^ In contrast to control cells showing an adherent monolayer, exposure of Sum190 cells to poly(I:C) for 4 days resulted in the formation of adherent spherical clusters with an increase in cluster size after continuous culture in the presence of poly(I:C). Notably, the majority of spherical clusters consisted of live cells (Figure 1a). These observations suggest that SUM190 cells can form a mammosphere-like structure (a property associated with CSCs) to survive and proliferate in response to poly(I:C) stimulation. Coincidently, the transcripts of *CD44* and *ALDH1* (two markers commonly used in characterization of breast CSCs) increased significantly after activation of TLR3. Furthermore, mRNA and protein levels of transcriptional factors associated with CSC properties^28^ (e.g., Nanog, Oct4, Sox2, Klf4, and c-Myc) increased markedly (Figures 1b and c). As such, these results point to a possible link between TLR3 activation and CSC phenotypes in SUM190 cells.

To determine whether the aforementioned observations are specific for TLR3, we examined TLR5, 7, and 8, which have also been implicated in enhancing antitumor immunity.^14^ Although SUM190 cells expressed TLR5, 7, and 8, the mammosphere-like structure and stem-like gene expression signatures were observed only after treatment with TLR3 ligand poly(I:C), but not with TLR5 ligand Flagenin or TLR7/8 ligand R848 (data not shown). This suggests that TLR3 activation uniquely associates with CSC-like properties in breast cancer cell SUM190.

To exclude the possibility that TLR3 activation is breast cancer cell-type specific, we also examined the breast ductal carcinoma cell line BT-483, adenocarcinoma cell line Cama-1 and triple-negative inflammatory breast cancer cell line SUM149. Triple-negative breast cancer and inflammatory breast cancer represent two of the most aggressive forms of human breast cancer, as characterized by their unique molecular profiles, aggressive behavior, and distinctive metastasis patterns.^29,30^ Significantly, we found that TLR3 expression was also present in SUM149, BT-483, and Cama-1 cells. Quantitative PCR (qPCR) results showed that poly(I:C)-treated cells exhibited a similar profile of gene expression upregulation associated with stem-cell properties and cancer metastatic potential. Specifically, CD44 and ALDH1, two important breast CSC markers, increased 2- to 17-fold in all cell lines examined (Figure 1d), suggesting that TLR3 activation, but not TLR5, 7, and 8, associates with CSC properties in different subtypes of breast cancer cells.

### TLR3 activation promotes CSC phenotypes *in vitro*

**Figure 2:**
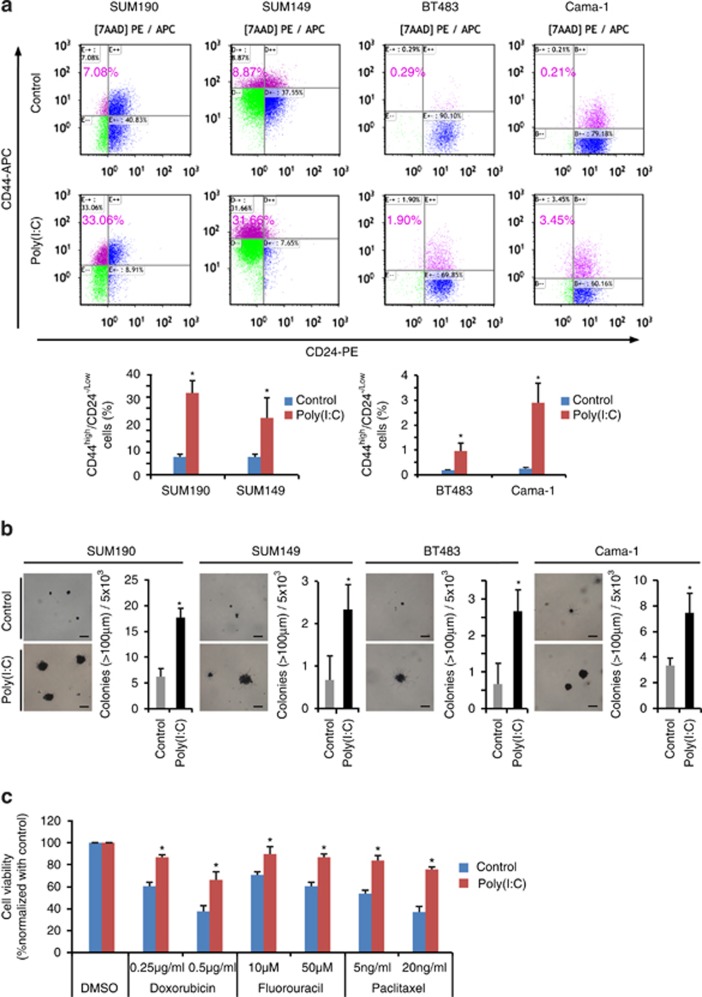
TLR3 activation promotes CSC phenotypes *in vitro.*(**a**) SUM190, SUM149, BT483, and Cama-1 breast cancer cells were grown in the absence or presence of 1 *μ*g/ml of poly(I:C) for 4 days. The percentage of CD44^high^/CD24^−/low^ cells was assessed using flow cytometry. (**b**) Soft-agar colony formation assays. All four breast cancer cell lines exhibit an increase in colony numbers (colony size >100 *μ*m) after stimulation with poly(I:C) in comparison with controls. Cells (5 × 10^3^/per well) were seeded in soft agar in 12-well plates for 17 days. Cellular aggregates with diameter of >100 *μ*m were evaluated as colonies after MTT staining for live cells. Scale bar, 100 *μ*M. (**c**) Cell viability of SUM190 in response to various chemotherapeutic compounds after treatment with vehicle or poly(I:C) for 2 days. Data represent the average±S.D. of three independent experiments. *P<0.05

Human breast CSCs are commonly characterized *in vitro* by expression of CD44^high^/CD24^−/low^ surface markers,^3,7,11^ tumorigenic capacity and drug resistance.^6,8,9^ Using flow cytometry (FACS), we first examine the changes in CD44^high^/CD24^−/low^ sub-population after poly(I:C) stimulation. Although non-stimulated SUM190 and SUM149 possess an inherently higher percentage of CD44^high^/CD24^−/low^ sub-population, poly(I:C) treatment led to a further upregulation of this subset by ~3- to 5-fold (Figure 2a). Similarly, a 6- and 16-fold boost of CD44^high^/CD24^−/low^ fraction was observed in poly(I:C)-treated BT483 and Cama-1 cell lines, respectively (Figure 2a).

To test whether tumorigenic potential is also affected by TLR3 activation, we performed soft-agar colony-forming assays and scored the numbers of colony (>100*μ*m). Anchorage-independent cell growth measured in the soft agar is the gold standard for cellular testing of potential therapeutic agents in oncology since the method was established by Hamburger and Salmon.^31^ This assay reflects the presence of self-renewing, gland-reconstituting stem cells within the population.^32^ As anticipated, we found that poly(I:C) treatment resulted in a >3-fold increase in colony-forming capacity than controls for SUM190 cells. A similar result was also obtained using SUM149, BT483, and Cama-1 cell lines with poly(I:C) treatment (Figure 2b ).

We further examined whether poly(I:C)-treated breast cancer cells became more resistant to chemotherapeutic drugs, another known CSC property.^8,9^ As expected, poly(I:C)-treated cells were more resistant than controls to two commonly used chemotherapeutic drugs, paclitaxel and doxorubicin (Figure 2c). Collectively, the significant increases in CD44^high^/CD24^−/low^ sub-population, clonogenic capacity, and drug resistance after poly(I:C) treatment demonstrate that TLR3 activation enhances breast CSC phenotypes *in vitro*.

We also observed that poly(I:C) promoted the growth of tumor cell aggregates and mammospheres (the methods used to enrich for cells with tumorigenic potential, Supplementary Figures S1 and S2). Moreover, poly(I:C) treatment significantly upregulated the expression of CSC markers in both fractionated CSC (CD44^high^/CD24^−/low^) and non-CSC (CD44^−/low^/CD24^high^) sub-populations (Supplementary Figure S3). It suggests that an increased pool of CSCs associates with the proliferation of CSCs along with the induction of CSC phenotypes from non-CSCs after TLR3 activation.

### TLR3 activation enriches breast CSCs *in vivo*.

**Figure 3:**
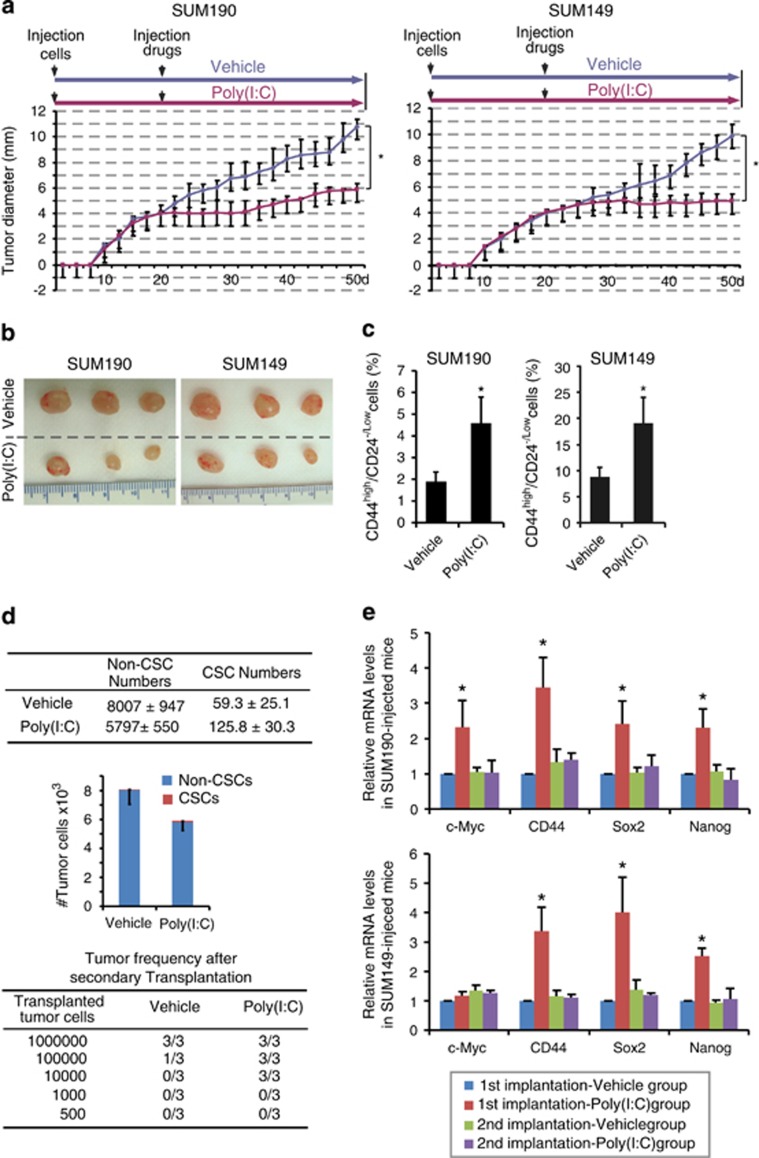
TLR3-activation enriches CSCs *in vivo*. (**a**) SUM190 and SUM149 cells were injected into inguinal fat pads of athymic nude mice. Mice were intraperitoneally given either vehicle or 250 *μ*g of poly(I:C) every other day after tumors reached an average size of 4 mm in diameter. Data represent the average±S.D., *n*=6–8 for SUM190, and 4–6 for SUM149; *P<0.01. (**b**) Tumors display a smaller size in poly(I:C)-treated mice compared with vehicle controls. (**c**) The percentage of CD44^high^/CD24^−/low^ cells in tumors was determined by flow cytometry. *n*=5; *P<0.05. (**d**) SUM190 xenografts harvested from the mice that had been injected with either vehicle or poly(I:C) after the first tumor cell transplantation were dissociated into single-cell suspensions and retransplanted into the mammary fat pad of new nude mice in serial limiting dilutions (1 000 000, 100 000, 10 000, 1000, or 500 cells per injection). CSC frequency was determined by flow cytometry. Data represent the average±S.D., *n*=3; *P<0.05. (**e**) Gene expression profiles of primary and secondary implanted tumor samples were determined using qPCR. mRNA levels of CSC-associated markers are increased only in primary tumor cells treated with poly(I:C). In the absence of continuous poly(I:C) injection in secondary transplantation, these changes have disappeared. Data represent the average±S.D., *n*=3; *P<0.05

To investigate whether TLR3 activation promotes induction of breast CSCs *in vivo*, we treated tumor-bearing mice with poly(I:C), and then analyzed the excised tumors for CSC-associated properties. The same number of SUM190 and SUM149 human breast cancer cells was implanted in the mammary fat pads of athymic nude mice. When tumors reached a mean diameter of 4 mm, control mice were intraperitoneally (i.p.) injected with vehicle while test mice with poly(I:C) (250 *μ*g every other day). As shown in Figures 3a and b, a decrease in tumor size was observed in poly(I:C)-treated mice in comparison with control group, indicating an inhibition in tumor growth. This result was consistent with a previous report that TLR3 agonists repress but not eradicate the established primary tumors.^33^ However, CD44^high^/CD24^−/low^ sub-population increased significantly by 2.2-fold in SUM190-transplanted mice and 2.3-fold in SUM149-transplanted mice (Figure 3c) after ~30 days of poly(I:C) administration. Consistently, absolute numbers of CD44^high^/CD24^−/low^ sub-population increased by 2.1-fold in SUM190-transplanted mice receiving poly(I:C) injection (Figure 3d), demonstrating that TLR3 activation retards tumor growth but enriches for breast CSCs.

To determine whether the tumors containing a larger fraction of CD44^high^/CD24^−/low^ sub-population induced by poly(I:C) administration possess greater tumor-initiating potential, we performed secondary transplantation. We serially diluted tumor cells containing various percentage of CD44^high^/CD24^−/low^ populations isolated from primary tumors and subsequently transplanted them into athymic nude mice without further poly(I:C) administration. Notably, tumor cells isolated from poly(I:C)-treated mice containing <3-fold higher numbers of CD44^high^/CD24^−/low^ cells exhibited a greater than 100-fold tumor-initiating capacity than control (Figure 3d; Supplementary Figure S4), suggesting a strong tumorigenic potential after poly(I:C) treatment.

We next tested expression level of several ‘stemness' genes in primary and secondary tumors. Primary tumors from poly(I:C)-treated mice bearing either SUM190 or SUM149 showed higher levels of CD44, Sox2, and Nanog expression compared with vehicle controls (Figure 3e). However, without further administration (i.e., a stimulation-withdraw) of poly(I:C), secondary tumors did not show an upregulation in those ‘stemness' genes (Figure 3e), indicative of a simulative effect of TLR3 activation on the induction and maintenance of breast CSC properties. Collectively, the results from FACS, secondary transplantation, and gene expression profiling demonstrate that TLR3 activation significantly enhances tumor-initiating capacity and promotes CSC phenotypes *in vivo*.

### NF-*κ*B signal is not exclusively responsible for TLR3 activation-enhanced CSC phenotypes

**Figure 4:**
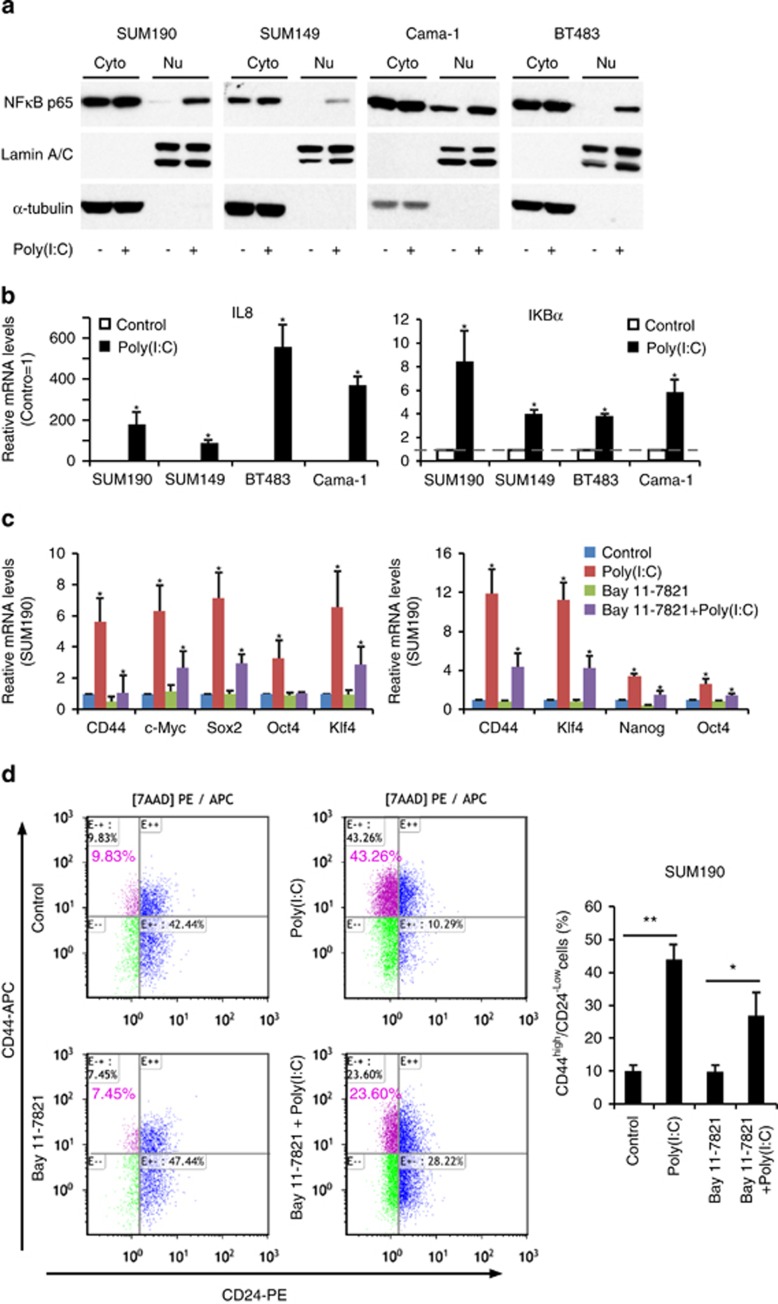
NF-*κ*B signal is not exclusively responsible for enhanced CSC phenotypes after TLR3 activation. (**a**) SUM190, SUM149, BT483, and Cama-1 cells were treated with 1 *μ*g/ml poly(I:C) for 4 days. Nuclear extracts (Nu) and cytoplasmic extracts (Cyto) were immunoblotted to examine nuclear translocation of NF-*κ*B p65. *α*-tubulin and Lamin A/C: internal loading controls for cytoplasmic and nuclear proteins, respectively. (**b**) qPCR analysis of target genes of NF-*κ*B pathway: IL8 and I*κ*B*α* in SUM190, SUM149, BT483, and Cama-1 following 1 *μ*g/ml of poly(I:C) treatment for 4 days. Data represent the average±S.D., *n*=3; *P<0.05. (**c**) qPCR analysis of the indicated CSC-associated genes. Cells were treated with NF-*κ*B inhibitor Bay 11-7821 (SUM190:5 *μ*M; SUM149:7.5 *μ*M) or vehicle (control) for 4 h and then co-treated with 1 *μ*g/ml of poly(I:C) or vehicle for 4 days. The expression levels of CSC-associated genes are decreased but not completely abolished after NF-*κ*B inhibition. Data represent the average±S.D., *n*=3; *P<0.05. (**d**) Flow-cytometry analysis of SUM190 CD44^high^/CD24^−/low^ sub-population after the same treatment as described in (**c**). Data represent the average±S.D., *n* =3; *P<0.05; **P<0.01

It is well known that cellular responses to TLR3 activation are principally regulated by NF-*κ*B signal pathway.^14^ As NF-*κ*B activates prosurvival pathways, we originally hypothesized that NF-*κ*B signaling was the key contributor to TLR3 activation-induced CSC phenotypes. We indeed observed a significant increase in nuclear translocation of NF-*κ*B p65 in all four breast cancer cell lines after poly(I:C) treatment (Figure 4a). The increased NF-*κ*B activities after TLR3 agonist stimulation were further confirmed by qPCR analysis, showing a higher expression in NF-*κ*B-dependent genes IL8 and I*κ*B*α* (Figure 4b).

**Figure 5:**
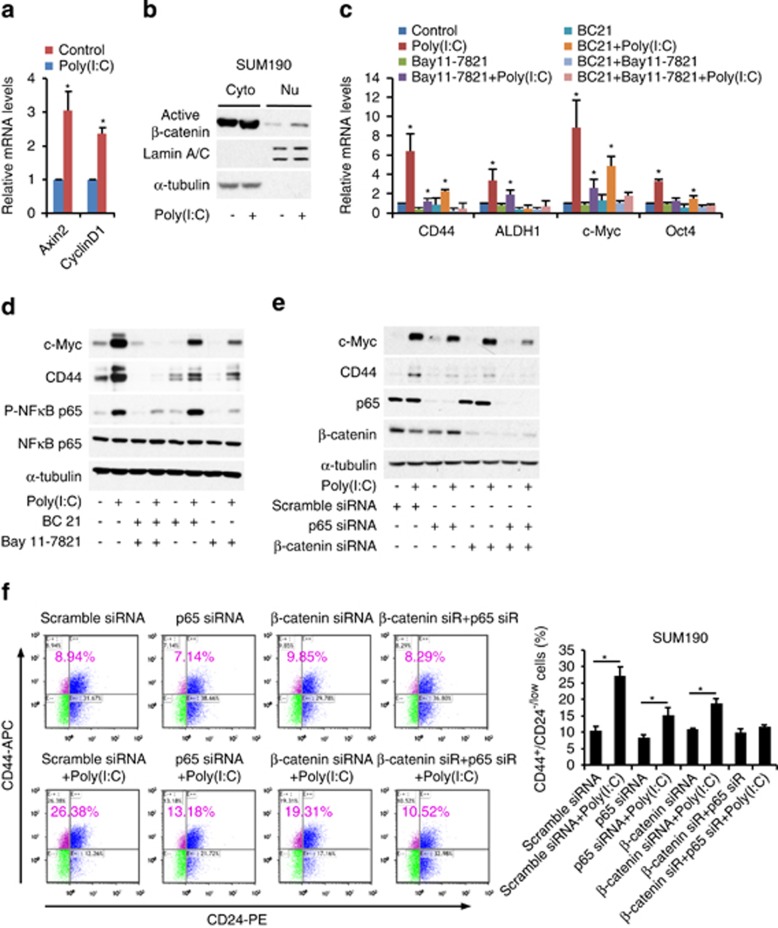
*β*-Catenin and NF-κB co-mediate TLR3 activation-induced CSC phenotypes. (**a**) qPCR analysis of target genes of *β*-catenin pathway (Axin2 and CylinD1) in SUM190 after treatment with 1 *μ*g/ml of poly(I:C) for 4 days. Data represent the average±S.D., *n*=5; *P<0.05. (**b**) Western blot analysis of nuclear translocation of active *β*-catenin in SUM190 cells after treatment with poly(I:C) (1 *μ*g/ml ) for 4 days. *α*-tubulin and Lamin A/C: internal loading controls for cytoplasmic (Cyto) and nuclear (Nu) proteins. (**c**) qPCR analysis of the indicated stem markers. SUM190 cells were pretreated for 4 h with Bay 11-7821 (5 *μ*M, an NF-*κ*B inhibitor) and BC21 (7.5 *μ*M, a *β*-catenin/TCF4 inhibitor) either alone or in combination, followed by stimulation with poly(I:C) for 4 days in the presence or absence of the above inhibitors. Data represent the average±S.D., *n*=3; *P<0.05. (**d**) Inhibition of both NF-*κ*B and *β*-catenin pathways completely blocks poly(I:C)-induced expression of CD44 and c-Myc (western blot). SUM190 cells were treated in the same way as those described in (**c**). (**e**) SUM190 cells were transfected with siRNA oligos against NF-*κ*B p65 (p65), *β*-catenin or non-targeting oligos for 24 h, and then treated with 1 *μ*g/ml of poly(I:C) for 3 days. Immunoblotting shows an almost complete knockdown of NF-*κ*B and *β*-catenin expression after co-transfection of NF-*κ*B p65 and *β*-catenin siRNA. (**f**) Knockdown of both NF-*κ*B (p65) and *β*-catenin abrogates an increase in CD44^high^/CD24^−/low^ cell population following poly(I:C) treatment. Data represent the average±S.D., *n*=3; *P<0.05

To confirm the role of NF-*κ*B in TLR3 activation-enhanced CSC phenotypes, we blocked NF-*κ*B pathway with a specific inhibitor BAY11-7082. Unexpectedly, NF-*κ*B inhibition was incapable of effectively blocking the increase in gene expression of CD44, c-Myc, Sox2, and Klf4 that associate with CSC properties (Figure 4c). FACS analysis also showed an ineffective repression on the elevated CD44^high^/CD24^−/low^ sub-population induced by TLR3 activation after NF-*κ*B inhibition with BAY11-7082 (Figure 4d). We further employed NF-*κ*B siRNA knockdown and obtained similar results (Figure 5f). Collectively, these data demonstrate that the NF-*κ*B signaling pathway is not exclusively responsible for TLR3 activation-enhanced CSC phenotypes in breast cancer cells.

### Combination of *β*-catenin and NF-*κ*B pathways is responsible for TLR3 activation-enhanced CSC phenotypes

To determine which signaling pathways co-contribute to the increased CSC phenotypes induced by TLR3 activation, we examined a number of pathways and finally focused on Wnt/*β*-catenin. *β*-Catenin has been implicated in different stages of mammary gland development and mammary oncogenesis.^34^ Cancer cells with increased *β*-catenin expression have been shown to exhibit greater metastatic potential.^35^ However, it is unknown whether *β*-catenin signaling is involved in TLR stimulation-enhanced CSC phenotypes. Here, we observed an upregulation of *β*-catenin targeted genes, Axin2 and cyclinD1, upon poly(I:C) treatment (Figure 5a), indicative of the activation of Wnt/*β*-catenin signaling pathway. Western blotting also showed an increase in nuclear translocation of active *β*-catenin after poly(I:C) treatment (Figure 5b). Specifically, using a *β*-catenin/TCF/LEF-dependent enhanced green-fluorescent protein (eGFP) reporter, we found that TCF-eGFP reporter activity was significantly increased in the lentivirus-transduced SUM190 cells after poly(I:C) treatment (Supplementary Figure S5). In combination with aforementioned observations, it suggests that TLR3 stimulation is capable of activating not only NF-*κ*B but also Wnt/*β*-catenin signaling in breast cancer cells.

To further determine whether *β*-catenin signal is involved in TLR3-induced CSC phenotypes, we used a *β*-catenin pathway inhibitor BC21. As shown in Figure 5c, BC21 only partially inhibited poly(I:C)-induced expressions of CSC-associated genes CD44, ALDH1, c-Myc, and Oct4. Strikingly, simultaneously inhibiting both NF-*κ*B and *β*-catenin pathways robustly suppressed TLR3 activation-induced upregulation of CSC-associated genes and proteins (Figures 5c and d). These observations were further confirmed by double knockdown of both NF-*κ*B p65 and *β*-catenin pathways since the expression levels of CD44 and c-Myc proteins were effectively suppressed (Figure 5e, western blot). Moreover, double knockdown of NF-*κ*B p65 and *β*-catenin, but neither alone, led to an almost complete abolition of the enriched CD44^high^/CD24^−/low^ sub-population induced by poly(I:C) stimulation (Figure 5f, FACS). Together, these data demonstrate that *β*-catenin and NF-*κ*B signals co-contribute to TLR3 activation-induced CSC phenotypes.

### Cardamonin is capable of blocking both *β*-catenin and NF-*κ*B pathways and abrogating TLR3 activation-enhanced CSC phenotypes *in vitro*

**Figure 6:**
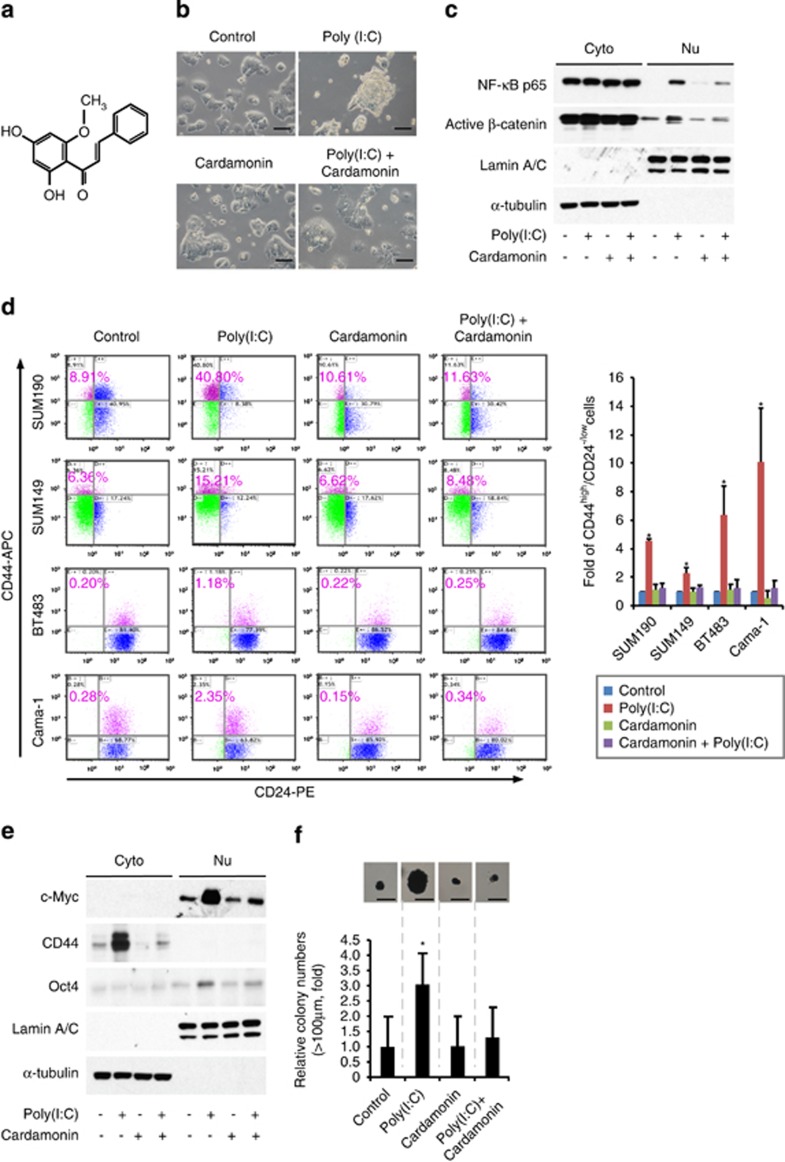
Cardamonin blocks both *β*-catenin and NF-*κ*B pathways and abrogates TLR3-activation-induced CSCs *in vitro*. (**a**) Molecular structure of cardamonin (2,4-dihydroxy-6-methoxychalcone) (**b**) Representative images of SUM190 cells treated with 1 *μ*g/ml of poly(I:C) in the presence or absence of cardamonin (10 *μ*M) for 4 days. Cardamonin abrogates the formation of non-adherent spherical clusters induced by poly(I:C) stimulation. Scale bars, 100 *μ*m. (**c**) Western blot analysis of nuclear translocation of NF-*κ*B p65 and active *β*-catenin. SUM190 cells were pretreated with 10 *μ*M of cardamonin for 4 h, followed by stimulation with poly(I:C) (1 *μ*g/ml) for 4 days in the presence of cardamonin. *α*-tubulin and Lamin A/C: internal loading controls for cytoplasmic (Cyto) and nuclear (Nu) proteins, respectively. (**d**) Flow-cytometry analysis of CD44^high^/CD24^−/low^ sub-population in four different breast cancer cell lines treated as described in (**c**). Data represent the average±S.D., *n*=3; *P<0.05. (**e**) Western blot analysis of c-Myc, CD44, and Oct4 proteins for the indicated groups as shown in (**c**). *α*-tubulin and Lamin A/C were included as internal loading controls for cytoplasmic (Cyto) and nuclear (Nu) extracts, respectively. (**f**) Soft-agar colony formation assay to evaluate tumorigenic potential. SUM190 cells were treated as described in (**c**), and seeded in soft agar in 12-well plates (5 × 10^3^ cells/well) for 17 days. Cellular aggregates with a diameter of >100 *μ*m were evaluated as colonies after staining with MTT for live cells. Data were expressed as fold increase over the control value (which was arbitrarily set to 1). Scale bar, 100 *μ*m. Data represent the average±S.D., *n*=4; *P<0.05

To identify compounds that can block breast CSC phenotypes promoted by TLR3 activation with clinical application potential, we screened a series of small molecular inhibitors targeting *β*-catenin and/or NF-*κ*B signals, and finally focused on cardamonin (Figure 6a). Cardamonin is one of the main ingredients from the seeds of Alpinia katsumadai Hayata and belongs to chalcone with antibacterial and anti-inflammatory effect. Significantly, cardamonin was capable of suppressing poly(I:C)-induced formation of mammosphere-like structure (Figure 6b) and inhibiting nuclear translocation of both NF-*κ*B and active *β*-catenin (Figure 6c). Western blot analysis of these tumor cells showed that cardamonin inhibited the expression of Oct4, c-Myc, and CD44 induced by poly(I:C) stimulation (Figure 6e). Furthermore, cardamonin almost completely abrogated the enriched CD44^high^/CD24^−/low^ CSC sub-population induced by poly(I:C) treatment in all four cell lines examined, including SUM190, SUM149, Cama-1, and BT483 (Figure 6d). Consistently, cardamonin treatment also significantly suppressed *in vitro* tumorsphere-forming capacity (Figure 6f). Together, the above data demonstrate that cardamonin abrogates TLR3 stimulation-induced CSCs *via* inhibiting both *β*-catenin and NF-*κ*B pathways.

### Cardamonin abolishes tumor-initiating potential induced by poly(I:C) *in vivo*

**Figure 7:**
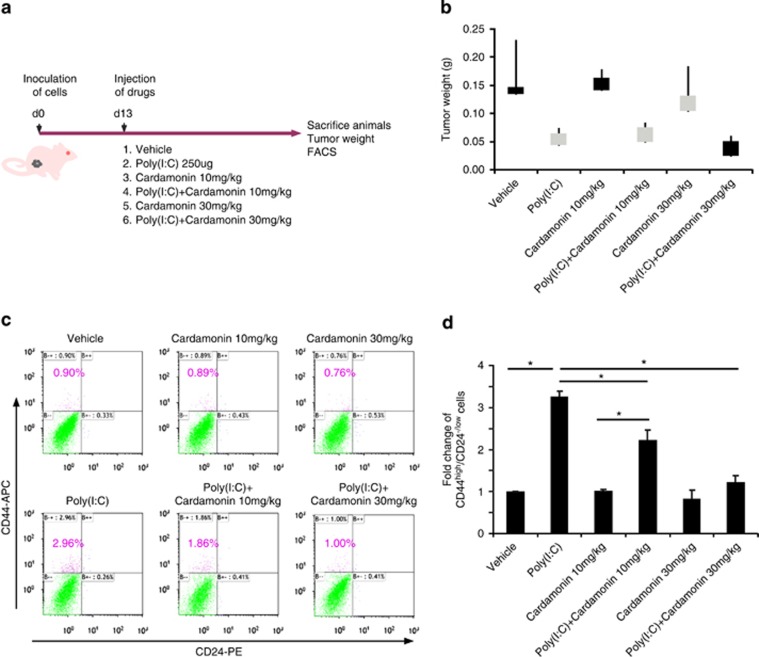
Cardamonin abolishes tumor-initiating potential induced by poly(I:C) *in vivo*. (**a**) Schematic diagram of *in vivo* experimental approaches. Mammary fat pads of nude mice were inoculated with SUM190 cells. Once tumors reached an average size of 4 mm in diameter, the mice were randomized into six treatment groups as shown in the diagram. Poly (I:C) and/or cardamonin were injected intraperitoneally every other day. (**b**–**d**) Although poly(I:C) injection retards tumor growth (**b**, tumor weight), it also significantly increases CSC pool. In contrast, co-injection of cardamonin abrogates poly(I:C)-enhanced CSC frequency (**c**), flow-cytometry analysis of CD44^high^/CD24^−/low^ sub-population) and total CSCs in each tumor (**d**). Data represent the average±S.D., *n*=3–4; *P<0.05

To determine whether cardamonin is capable of repressing poly(I:C)-enhanced CSCs *in vivo*, we implanted SUM190 cells in the mammary fat pads of athymic nude mice followed by drug treatment. When the tumor reached a mean diameter of 4 mm, mice were randomized into six groups and injected i.p. with poly(I:C) and/or vehicle plus 10 or 30 mg/kg of cardamonin every other day (Figure 7a). Tumor volumes were measured every other day after drug treatment. On day 32, animals were killed and tumors were harvested and weighed. An increase in the CSC pool was observed in the mice treated with poly(I:C) alone though tumor weights were lower than vehicle control (Figures 7b–d). Importantly, 30 mg/kg of cardamonin treatment almost completely abolished poly(I:C) stimulation-induced CSCs in mouse xenografts (Figures 7c and d). Given no substantial side effects observed after cardamonin injection every other day for 20 days, it is possible to further increase injection frequency and/or dosages to optimize treatment efficacy. Collectively, cardamonin abrogates poly(I:C)-induced CSCs not only *in vitro* (Figure 6) but also *in vivo* (Figure 7) and can be considered as a potential drug candidate to prevent the TLR3 activation-enriched breast CSCs.

## Discussion

Recurrence of breast cancer remains a significant clinical challenge. Inconsistent clinical trials using TLRs to boost immune systems against cancer is largely due to our incompletely understanding of TLRs' roles in CSCs. In this study, we demonstrate that TLR3 activation leads to breast CSC enrichment *in vitro* and *in vivo*. It is intriguing that a combination of NF-*κ*B and *β*-catenin signals, but neither alone, is fully responsible for poly(I:C)-enhanced CSC phenotypes. Cardamonin, a small molecule identified in this study, is capable of blocking both NF-*κ*B and *β*-catenin pathways to effectively abolish TLR3 activation-enriched CSCs *in vitro* and *in vivo* (Supplementary Figure S6).

### An unappreciated role of TLR3 in enriching breast CSCs

The importance of TLR3 signaling in CSC phenotypes has not been established until now. In early efforts, several studies found that activation of TLR3 signal displayed anticancer properties, possibly mediated by IFN*γ* production and/or TLR3-induced apoptosis.^33,36^ Others reported that elevated TLR3 expression in breast cancer patients was associated with poor prognosis.^22^ These findings suggest that TLR3 has more complicated biological functions than previously understood. In this study, we found that TLR3 activation did induce certain tumor cell death while concurrently potentiating CSC phenotypes and tumor-initiating capacity in breast cancer cells. Using qPCR analysis and soft-agar colony-forming assays, we showed a significant upregulation of various stem cell markers and an increase in tumor propagating properties *in vitro* after treatment with TLR3 agonist poly (I:C). Our secondary implantation model further revealed that TLR3 agonist markedly promoted tumor growth in nude mice. Hence, despite an initial growth retardation after TLR3 activation, the acquisition of CSC phenotypes in the remaining tumor cells could engender a stronger and more robust ‘second wave' of tumor growth (greater than 100-fold, Figure 3d). On the basis of our results that TLR3 activation hinders tumor growth but enriches breast CSCs, the examination of tumor size (or total number of cancer cells) instead of CSC sub-populations could lead to a different conclusion.

### *β*-Catenin signaling is required for TLR3 activation-enhanced breast CSC phenotypes

A direct connection among TLR3, *β*-catenin signaling, and breast CSCs was previously lacking. TLR3 signaling is known to culminate in activation of transcriptional factor NF-*κ*B, which controls the expression of a battery of pro-inflammatory genes. NF-*κ*B has also been considered to be a key contributor in promoting tumor progression, as it is found to be frequently overactivated in human breast cancers.^37,38^ However, a moderate suppression of breast CSC phenotypes following blockage of TLR3-mediated NF-*κ*B activation, as shown in our results, suggests other pathways also take place.

Here, we provide the first evidence that *β*-catenin, unexpectedly, is essential for TLR3 activation-enhanced breast CSC phenotypes. Significantly, after knockdown of both NF-*κ*B and *β*-catenin, the enrichment in CSC population was almost entirely lost. This emphasizes that co-activation of both NF-*κ*B and *β*-catenin, but neither alone, following TLR3 stimulation leads to the acquisition of a CSC phenotype. Given that poor clinical prognosis was associated with elevated TLR3 expression in breast cancer,^22^ it is possible that tumor cells with high levels of TLR3 expression and activation of NF-*κ*B and Wnt/*β*-catenin pathways are more resistant to conventional therapies and easy to relapse. Hence, understanding how CSCs are regulated by TLR3 stimulation and *β*-catenin activation is crucial if they are to be targeted for therapy.

### TLR3 agonists and CSCs

A triad of TLR3 stimulation, NF-*κ*B and *β*-catenin activities in promoting CSC phenotypes indicates a combinational use of TLR3 agonists with NF-*κ*B and *β*-catenin inhibitors, but neither alone, may help to improve clinic outcomes and efficacy of TLR3 agonists in patients with breast cancer. In an effort to introduce new approaches to target CSCs, we screened a number of small molecules, and identified cardamonin, a chalcone isolated from the fruits of a local plant. Cardamonin treatment effectively blocking TLR3 activation-induced CSCs *in vitro* and in a mouse xenograft model suggests its strong potential and high specificity of targeting breast CSCs. In particular, since elevated TLR3 expression was also highly expressed in other cancer patients and reversely correlated with poor prognosis, it is possible that inhibition of tumor TLR3 pathways may facilitate cancer treatment. We believe that the findings presented here broaden our understandings of the roles of TLR3, *β*-catenin and NF-*κ*B in CSC biology, and also have potential clinical implications in terms of preventing induction of CSC phenotypes and targeting cancer stem-like cells under inflammatory conditions and endogenous stimuli of damaged cellular components.

## Materials and Methods

### Cell culture and reagents

SUM190 and SUM149 were obtained from Asterand (Detroit, MI, USA) and cultured in Hams F-12 media (Mediatech, Manassas, VA, USA) containing 5 *μ*g/ml insulin, 1 *μ*g/ml hydrocortisone, antibiotics (penicillin/streptomycin), and 5% (SUM149) or 2% (SUM190) of fetal bovine serum (FBS) (HyClone, Logan, UT, USA). Medium for SUM190 cells was further supplemented with 5 mM ethanolamine, 10 mM HEPES, 5 *μ*g/ml transferrin, 6.6 ng/ml 3,3',5-triiodo-L-thyronine sodium salt, 8.7 ng/ml sodium selenite, and 1 mg/ml bovine serum albumin (BSA). Cells were cultured at 37 °C in a 5% CO_2_ incubator. Breast cancer cell lines BT483 and Cama-1 were purchased from the American Type Culture Collection (Manassas, VA, USA) and maintained in DMEM-F12 (1:1) supplemented with 10% FBS. Poly(I:C) was obtained from InvivoGen (San Diego, CA, USA). Doxorubicin hydrochloride, 5-Fluorouracil, Paclitaxel, Insulin, Hydrocortisone, HEPES, and BSA were purchased from Sigma-Aldrich (St. Louis, MO, USA). Cardamonin and Bay 11-7821 were purchased from TOCRIS Bioscience (Ellisville, MO, USA).

### Transfection, transduction, and *β*-catenin/TCF-eGFP reporter assays

The *β*-catenin/TCF/LEF-dependent reporter plasmid (7 × Tcf-eGFP/SV40-PuroR, 7TGP) was provided by Dr. Nusse *via* Addgene.^39^ Lentiviral production was carried out as described previously.^40^ Briefly, ten 10-cm dishes were seeded with 6 × 10^6^ cells per dish overnight before transfection. For two dishes, 8 *μ*g of the lentiviral vector, 5.4 *μ*g of the psPax2 envelope plasmid, 3.6 *μ*g of the packaging plasmid (pMD2.G) were used. The medium was replaced overnight, and lentiviral supernatant was harvested after 48 hours, filtered through a 0.45-*μ*m PES filter, and concentrated with Lenti-X concentrator (Clontech, Mountain View, CA, USA). For viral infection, once SUM190 cells in 6-well plate reached 40–50% confluence, 1 ml of concentrated lentiviral supernatant and 8 *μ*g/ml of polybrene were added for 24 h. The infected cells containing the reporter TCF-eGFP cassette were selected with puromycin. The TCF-eGFP expression levels after poly(I:C) treatment for 6, 24, and 48 h were determined by FACS.

### Xenograft tumor growth

All mouse experimentation was conducted in accordance with standard operating procedures approved by the Animal Care Committee at the University of Ottawa. Athymic nude mice (6 weeks old, female, 20–22 g body weight) were obtained from Charles River Laboratories (Saint-Constant, QC, Canada). To establish breast cancer xenografts in nude mice, SUM190 or SUM149 cells were mixed with Matrigel and injected under aseptic conditions into mammary fat pads of nude mice (*n*=3–8 for each group, 1 × 10^6^ cells per fat pad). The tumor was monitored and evaluated every 2–3 days with calipers. Tumors were measured in two dimensions, and volume was calculated according to the formula: *V*=0.5 × (length) × (width).^2^ When tumors reached a mean diameter of 4 mm, tumor-bearing mice were randomly assigned to six treatment groups: (1) vehicle (control), (2) poly(I:C) (250 *μ*g), (3) cardamonin (10 mg/kg), (4) cardamonin (10 mg/kg) plus 250 *μ*g of poly(I:C), (5) cardamonin (30 mg/kg), and (6) cardamonin (30 mg/kg) plus 250 *μ*g of poly(I:C). Mice were injected i.p. with the drugs every other day. At the end of drug treatment, mice were humanely euthanized, and tumors were collected, weighed, disaggregated, and further analyzed by FACS and qPCR.

### Secondary transplantation of nude mouse model

Tumor tissues were dissociated mechanically and enzymatically to obtain a single-cell suspension. Tumors were minced by scalpel and incubated in Hams F-12 (Invitrogen, Carlsbad, CA, USA) containing collagenase/hyaluronidase (STEMCELL Technologies, Vancouver, BC, Canada) at 37 °C for 60 min. The tissues were further dissociated by pipette trituration and then passed through a 40-*μ*m nylon mesh to produce a single-cell suspension. Two groups of mice were implanted with tumor cells. Each mammary fat pad of athymic nude mouse was inoculated with 1 000 000, 100 000, 10 000, 1000, or 500 tumor cells harvested from mice that had been injected i.p. with vehicle or poly(I:C) after the first tumor cell transplantation. Tumor size was measured every other day.

### FACS analysis

Cancer cells dissociated from transplanted tumor tissues or from culture plates were counted and resuspended in 100 *μ*l of HBSS containing 2% heat-inactivated FBS (HIFS) and 10^5^ cells. Five microliters of mouse IgG solution (1 mg/ml) was added and incubated on ice for 10 min. According to the manufacturer's recommendation, appropriate antibodies were added and incubated for 30 min on ice. Then, cells were washed twice with HIFS and resuspended in 0.2 ml of HIFS that contained 7-aminoactinomycin D (7-AAD, 1 *μ*g/ml final concentration). Antibodies used were anti-CD44 (APC) and anti-CD24 (PE), which were purchased from BD Pharmingen (San Diego, CA, USA). Dead cells were eliminated by using viability dye 7-AAD. FACS was performed on a Cyan-ADP 9 (Beckman Coulter, Brea, CA, USA).

### Fractionation of CSC and non-CSC sub-populations from breast cancer cells

To separate CSCs from non-CSCs (SUM190 and Cama-1 cancer cells), single-cell suspensions were stained with CD44 antibody (APC-conjugated) and CD24 antibody (PE-conjugated) for 30 min, analyzed and sorted by FACS. CSCs are defined by the minority CD44^high^/CD24^−/low^ population, whereas non-CSCs are defined by the majority CD44^−/low^/CD24^high^ population. The CSCs and non-CSCs populations were sorted again to increase their purity (>99.2% in all cases). CSCs were cultured in serum-free special medium. Non-CSCs were cultured overnight in DMEM-F12 containing 2% FBS to allow cell attachment and survival. The medium was changed to serum-free special medium next day to keep the experimental conditions as same as CSCs.

### Soft-agar colony formation

SUM190, SUM149, BT-483, and Cama-1 cells were cultured in the presence of poly(I:C) or reagents as indicated for 4 days. A soft-agar assay was performed on 12-well plates with a base layer of 0.5% agarose gel containing DMEM. To generate the cell layer, 5 × 10^3^ cells/well were suspended in 0.35% top agarose gel in DMEM/F12 medium containing B27 supplement, 20 ng/ml of EGF, and 20 ng/ml of basic FGF. Plates were incubated at 37 °C in 5% CO_2_ for 17 days to allow colony formation, and cell viability was determined by staining with 3-(4,5-dimethylthiazol-2-yl)-2,5-diphenyl tetrazolium bromide (MTT, 1 mg/ml). Colonies of each cell line were counted (>100 *μ*m in diameter). All experiments were performed in triplicate, and data are presented as means±S.D.

### Mammosphere formation assay

Sum190 were cultured in the presence or absence of 1 *μ*g/ml of poly(I:C) for 4 days, dissociated into single-cell suspension and reseeded on ultra-low attachment plates at a density of 1 × 10^3^ cells/well (12-well plate) in DMEM/F12 medium containing B27 supplement, 20 ng/ml of EGF, and 20 ng/ml of basic FGF. Plates were incubated at 37 °C in 5% CO_2_ for 4 days to allow mammosphere formation followed by qPCR analysis.

### siRNA knockdown

siRNAs for NF-*κ*B p65 and *β*-catenin, and control scrambled siRNA were purchased from Thermo Scientific Dharmacon (Thermo Scientific, Rockford, IL, USA) as SMARTpools. For siRNA transfections, cells were transiently transfected with these oligos using Lipofectamine RNAiMAX reagent (Invitrogen) according to the manufacturer's instructions. Transfection efficiency was assessed using qPCR and western blot. One *μ*g/ml of poly(I:C) was added on the second day after siRNA transfection. After transfection for 72 h, cells were trypsinized and subjected to qPCR and FACS assays.

### Western blot analysis

For western blot analysis, cells were harvested and prepared using RIPA buffer (Sigma-Aldrich) and subcellular fractions taken using the NE-PER Nuclear Protein Extraction Kit (Thermo Scientific) containing protease inhibitor cocktails (Roche, Mannheim, Germany). Protein concentration was determined using a Bio-Rad DC protein assay kit (Bio-Rad, Hercules, CA, USA). Subsequently, 25–30 *μ*g of total protein was loaded onto an 8–12% SDS-polyacrylamide gel for electrophoresis and then transferred onto a PVDF membrane. Protein was identified by incubating the membrane with primary antibodies, followed by horseradish peroxidase-conjugated secondary antibodies and an enhanced chemiluminescence solution (Pierce, Thermo Scientific). Antibodies used in this study include: anti-CD44 (8E2) monoclonal antibody (5640), anti-Lamin A/C polyclonal antibody (2032), anti-c-Myc (D84C12) polyclonal antibody (5605), and anti-Sox2 (L1D6A2) monoclonal antibody (4900) from Cell Signaling (Danvers, MA, USA); anti-phospho-NF-*κ*B p65 pSer 536 monoclonal antibody (MA5-15160) from Thermo scientific; anti-Klf4 antibody (ab72543) from ABCAM (Toronto, ON, Canada); anti-Oct4 (N-19) polyclonal antibody (sc-8628) from Santa Cruz (Santa Cruz, CA, USA); anti-Nanog polyclonal antibody (AF1997) from R&D Systems (Minneapolis, MN, USA); anti-*β*-catenin (Clone 14, 610153) from BD (Mississauga, ON, Canada); anti-active *β*-catenin (anti-ABC, clone 8E7, 05665) from Millipore (MI, Billerica, MA, USA); anti-*α*-tubulin monoclonal antibody (T9026) from Sigma-Aldrich.

### Quantitative real-time PCR

Total RNAs were extracted using RNeasy kit (Qiagen, Valencia, CA, USA) and real-time qPCR analysis was performed using Bio-Rad MyiQ (Bio-Rad) as previously described.^41^ The conditions for qPCRs are one cycle at 95 °C for 20 s, followed by 40 cycles at 95 °C for 3 s and annealing at 60 °C for 30 s. Results were normalized to the housekeeping gene glyceraldehyde 3-phosphate dehydrogenase (GAPDH). Relative expression level of genes from different groups was calculated with the ^2ΔΔ^CT method and compared with the expression level of the corresponding gene in control cells. Specific primer sequences for individual genes are listed in Supplementary Table S1.

### Growth rates and cell viability assays

To measure cell growth rates, 1000 cells per well were seeded onto 96-well plates in triplicates. One day after seeding, compounds were added in five replicates per concentration for each cell line. After 48 h, MTT reagent (tetrazolium) was added for 4 h and the reaction was stopped by removing MTT and adding. DMSO (150 *μ*l) was added to each well to dissolve formazan crystals. Absorbance was measured at 570 nm. Cell viability was also assessed by a hemocytometer-based trypan blue dye exclusion.

### Statistical analyses

Data are expressed as mean±standard deviation (S.D.) unless specified elsewhere. Statistical significance was determined using a Student's *t*-test, or ANOVA wherever appropriate. Results were considered as significant when *P*<0.05.

## Abbreviations

CSCs: cancer stem cells
FBS: fetal bovine serum
FACS: flow cytometry
7-AAD: 7-aminoactinomycin D
qPCR: quantitative PCR
TLRs: toll-like receptors
TLR3: toll-like receptor 3
dsRNA: double-stranded RNA
poly(I:C): polyinosinic-polycytidylic acid
i.p.: intraperitoneally
MTT: 3-(4,5-dimethylthiazol-2-yl)-2,5-diphenyl tetrazolium bromide
GAPDH: glyceraldehyde 3-phosphate dehydrogenase
Cyto: cytoplasmic
Nu: nuclei
Cardamonin: 2,4-dihydroxy-6-methoxychalcone
eGFP: enhanced green-fluorescent protein
