# Supplementary material for: β-Catenin and NF-κB co-activation triggered by TLR3 stimulation facilitates stem cell-like phenotypes in breast cancer
Source: Cell Death Differ. 2014 Sep 26;22(2):298–310. doi: 10.1038/cdd.2014.145 (PMC4291491; doi:10.1038/cdd.2014.145)
Supplement: Supplementary Information [file cdd2014145x1.pdf]

Supplemental Information

β-catenin and NF-κB co-activation triggered by TLR3 stimulation facilitates stem cell-like phenotypes in breast cancer

Deyong Jia[1], Weixia Yang[1,2], Li Li[1], Huijuan Liu[1,3], Yuan Tan[1], Sarah Ooi[1], Lingyi Chi[4], Lionel G. Filion[1], Daniel Figeys[1,5,7], Lisheng Wang[1,6,7]

[1] Department of Biochemistry, Microbiology and Immunology, Faculty of Medicine, University of Ottawa, 451 Smyth Road, Ottawa, ON K1H 8M5, Canada
[2] Life Science College of Northwest A&F University, Yangling, Shaanxi712100, China
[3] Bio-X Institutes, Key Laboratory for the Genetics of Developmental and Neuropsychiatric Disorders, Ministry of Education, Shanghai Jiao Tong University, Shanghai 200240, China
[4] Department of Neurosurgery, Qilu Hospital of Shandong University, Jinan, Shandong 250012, China
[5] Department of Chemistry, University of Ottawa
[6] Regenerative Medicine Program, Ottawa Hospital Research Institute,
[7] Ottawa Institute of Systems Biology, University of Ottawa, Canada

* Correspondence: Lisheng Wang (lwang@uottawa.ca)

CONTENT OF SUPPLEMENTARY INFORMATION

I. SUPPLEMENTARY TABLE

Table S1, related to Materials and Methods
List of primers used in quantitative PCR for measuring gene expression relative to GAPDH.

II. SUPPLEMENTARY FIGURES

Figure S1, related to Figure 2
Prolonged poly(I:C) treatment further promotes the growth of tumor cell aggregates with increased expression levels of stem cell markers.

Figure S2, related to Figure 2
Poly(I:C)-treated breast cancer cells exhibit a higher capacity to form mammospheres.

Figure S3, related to Figure 2
The increased expressions of CSCs-markers in both $CD44^{high}/CD24^{-/low}$ and $CD44^{-/low}/CD24^{high}$ subpopulations after poly(I:C) treatment.

Figure S4, related to Figure 3
Representative photographs of the nude mice after secondary transplantation.

Figure S5, related to Figure 5
Poly(I:C) treatment activates Wnt/β-catenin signaling pathway.

Figure S6
A graphical outline.

I. SUPPLEMENTARY TABLE

Table S1. List of primers used in quantitative PCR for measuring gene expression relative to GAPDH (Related to Materials and Methods)

Gene names	Forward	Reverse
Oct4	CTGCAGTGTGGGTTTCGGGCA	CTTGCTGCAGAAGTGGGTGGAGGAA
Nanog	CATGAGTGTGGATCCAGCTTG	CCTGAATAAGCAGATCCATGG
IL-6	AACAACCTGAACCTTCCAAAGA	TCAAACTCCAAAAGACCAGTGA
Axin2	GTCACCAAACCCATGCCTGTCTCT	TAAGCACCGTCTTGATCGCCCAAT
Cyclin D1	AATGACCCCGCACGATT	GCACAGAGGGCAACGAAGG
IKBa	CTCCGAGACTTTCGAGGAAATAC	GCCATTGTAGTTGGTAGCCTTCA
IL-8	ATGACTTCCAAGCTGGCCGTGGCT	TCTCAGCCCTCTTCAAAAACTTCTC
Klf4	CAAGTCCCGCCGCTCCATTACCAA	CCACAGCCGTCCCAGTCACAGTGG
TLR3	TAGCAGTCATCCAACAGAATCAT	AATCTTCTGAGTTGATTATGGGTAA
MMP9	TTGACAGCGACAAGAAGTG	CTGAGGAATGATCTAAGCC
ALDH1	CGCAAGACAGGCTTTTCAG	TGTATAATAGTCGCCCCCTCTC
TERT	ATGCGACAGTTCGTGGCTCA	ATCCCCTGGCACTGGACGTA
CD44	AGACAACCACAAGGATGACTGATG	TCCAGTTTCCTTCATAAGCAGTGG
c-Myc	TTCTCTCCGTCCTCGGATTCTCTG	TCTTCTTGTTCCTCCTCAGAGTCG
Sox2	CATCACCCACAGCAAATGACAGC	TTGCGTGAGTGTGGATGGGATTG
GAPDH	ACAGTCAGCCGCATCTTCTT	GACAAGCTTCCCGTTCTCAG

II. SUPPLEMENTARY FIGURES

Figure S1

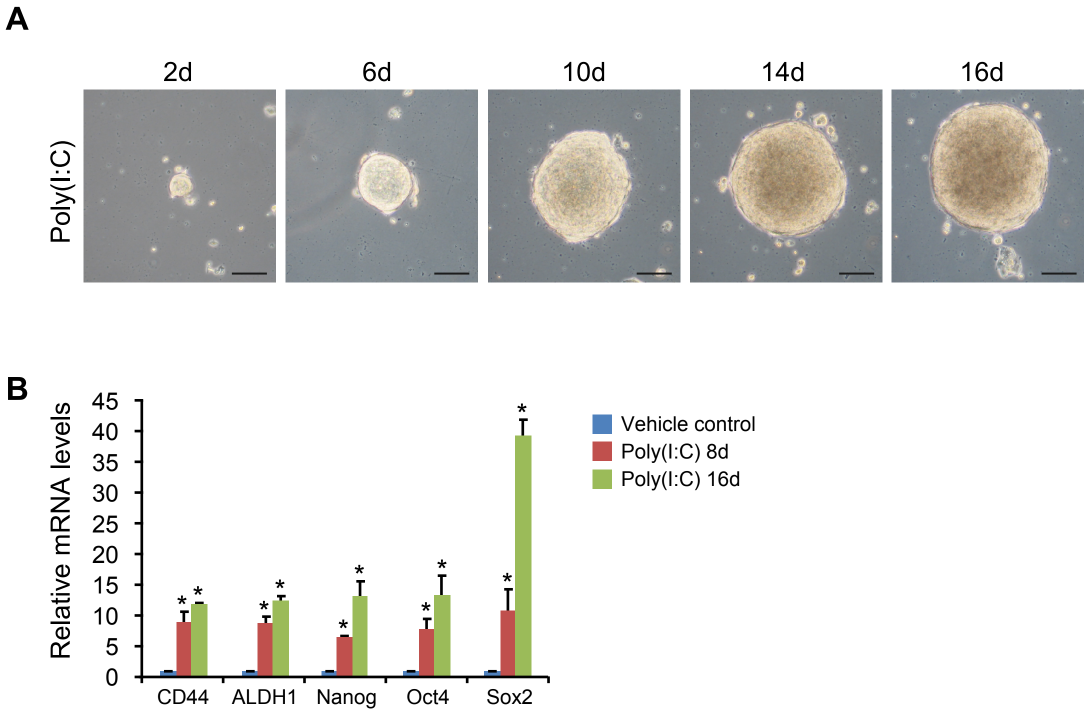

Figure S1. Prolonged poly(I:C) treatment further promotes the growth of tumor cell aggregates with increased expression levels of stem cell markers, related to Figure 2.

A. Phase-contrast images of the growing colonies after 1μg/ml of poly(I:C) treatment for 2d, 6d, 10d, 14d, and 16d. Scale bars, 100 μm.

B. Quantitative real-time PCR analyses of the indicated genes in SUM190 cells after treatment with 1μg/ml of poly(I:C) for representative days (8d and 16d). GAPDH mRNA was used to normalize variability in template loading. Data represent the average ± SD, n = 3; * $p < 0.05$

Figure S2

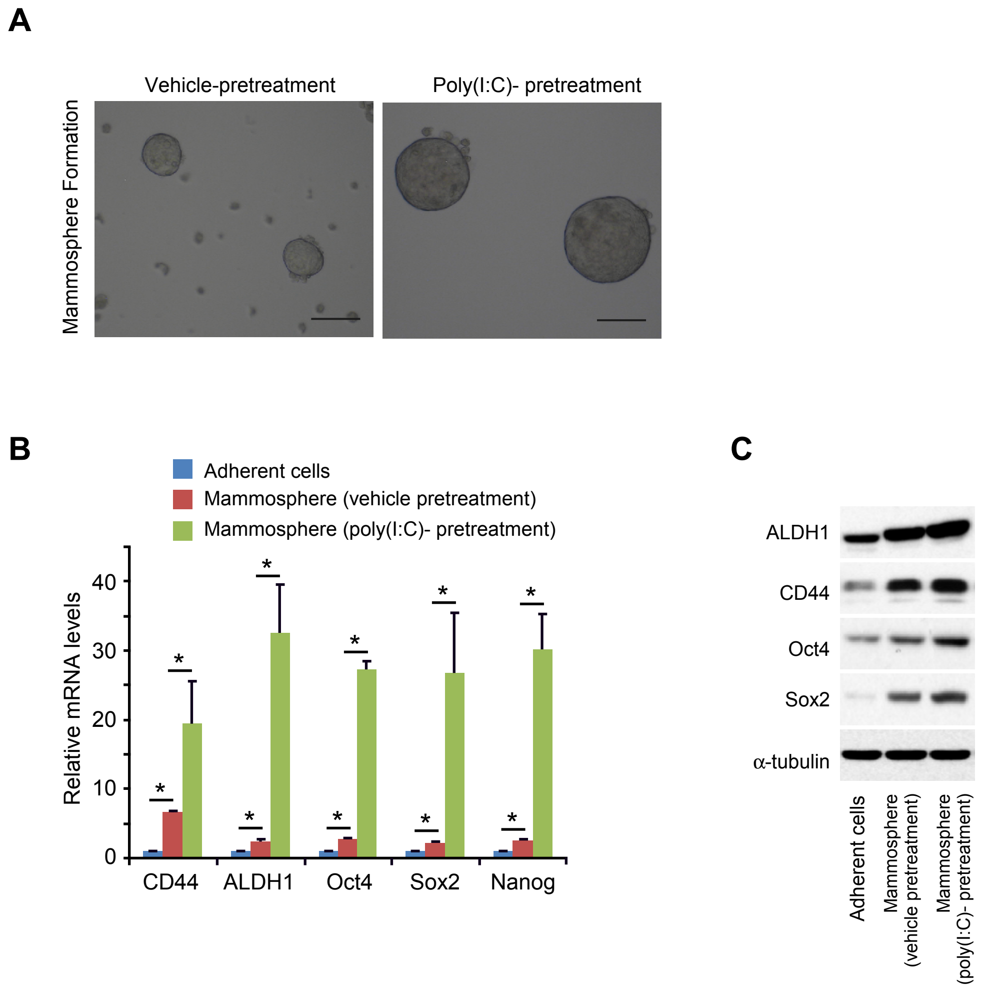

Figure S2. Poly(I:C)-treated breast cancer cells exhibit a higher capacity to form mammospheres, related to Figure 2.

A. SUM190 cells were treated with vehicle (control) or poly(I:C) for 4d and re-seeded in mammosphere culture conditions for 4d to assess their mammosphere formation capacity. Scale bars, 100 μm.

B. Quantitative real-time PCR analyses of the indicated genes in adherent cells, mammospheres after vehicle-pretreatment or poly(I:C)-pretreatment. GAPDH mRNA was used to normalize variability in template loading. Data represent the average ± SD, n = 3; * $p < 0.05$.

C. Western blot analysis of ALDH1, CD44, Oct4, and Sox2 for the indicated groups as shown in B. α-tubulin was included as an internal loading control.

Figure S3

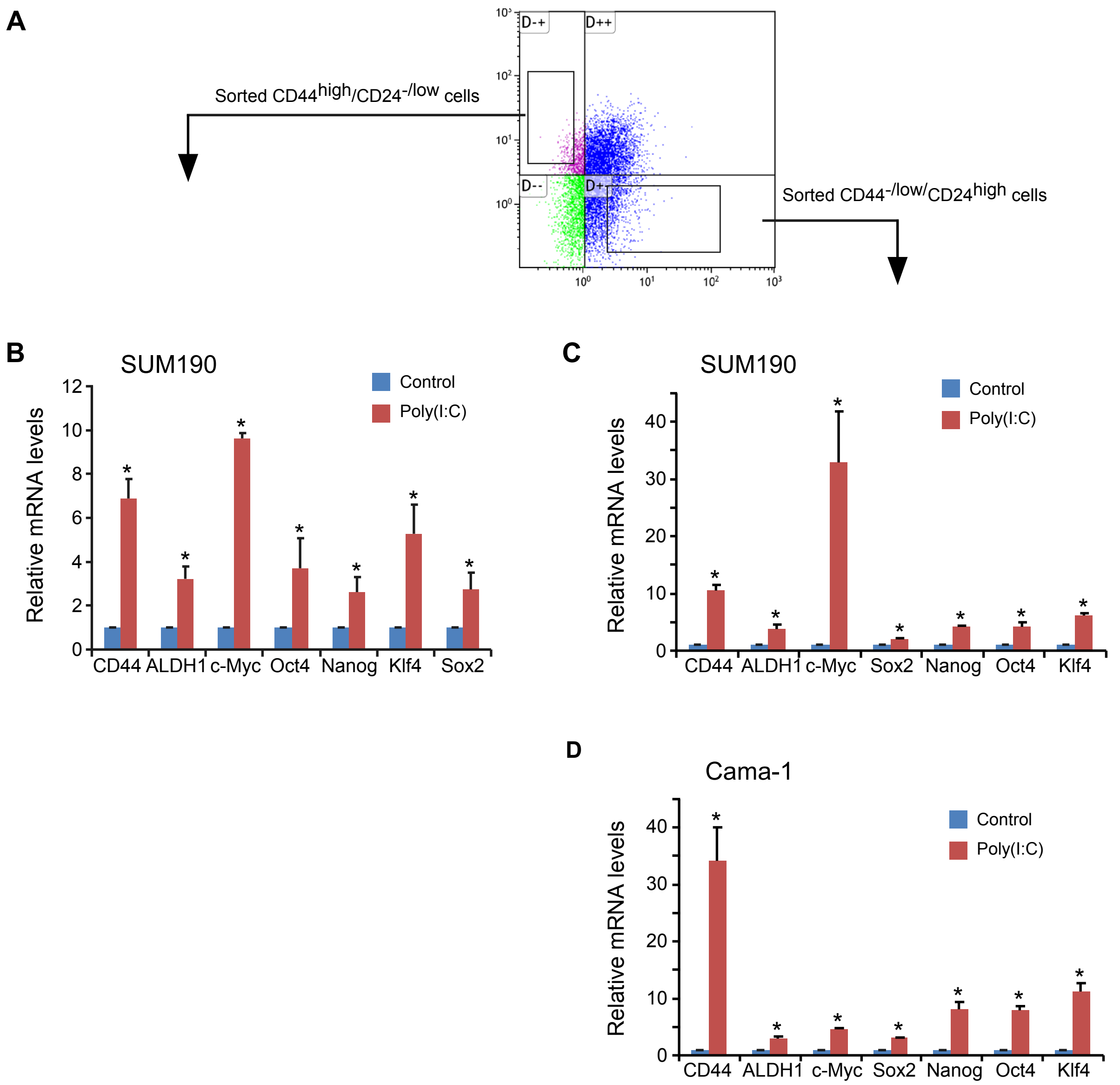

Figure S3. The increased expressions of CSCs-markers in both CD44^{high}/CD24$^{-/low}$ and CD44$^{-/low}$/CD24^{high} subpopulations after poly(I:C) treatment, related to Figure 2.

CD44^{high}/CD24$^{-/low}$ and CD44$^{-/low}$/CD24^{high} cells were fractionated by flow cytometry (**A**) and cultured in the medium containing 1μg/ml of poly(I:C) for 4d. Total RNA was extracted from the cells using the RNeasy Mini kit from Qiagen followed by quantitative real-time PCR analysis. The indicated stem markers were significantly up-regulated after poly(I:C) treatment in both CD44^{high}/CD24$^{-/low}$ (representing CSC subset; **B**, SUM190 breast cancer cells) and CD44$^{-/low}$/CD24^{high} cells (representing non-CSC subset; **C**, SUM190; **D**, Cama-1 breast cancer cells). These results suggest that poly(I:C) promotes CSC phenotypes in both CSC and non-CSC subpopulations. Data represent the average ± SD, n = 3; * $p < 0.05$.

Figure S4

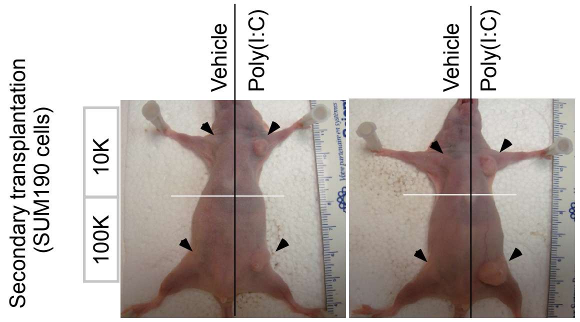

Figure S4. Representative photographs of the nude mice after secondary transplantation, related to Figure 3.

Each mammary fat pad of nude mice was inoculated with 100,000 or 10,000 of SUM190 cells harvested from the first transplanted mice that had been injected with vehicle or poly(I:C) for approximately 30d.

Figure S5

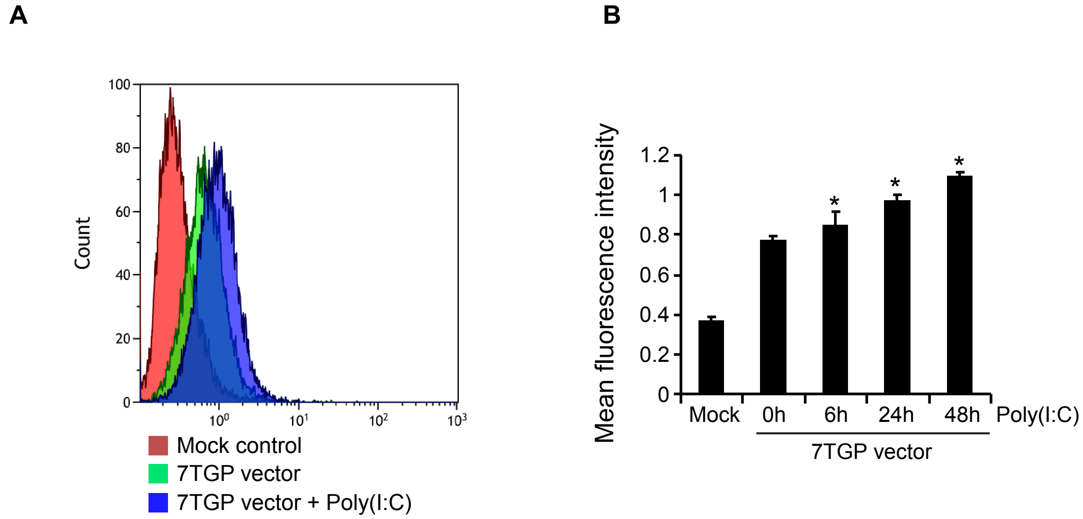

Figure S5. Poly(I:C) treatment activates Wnt/β-catenin signaling pathway, related to Figure5.

A. Flow cytometry analysis for the expression of TCF-eGFP reporter activity (7TGP vector) in the lentivirus-transduced SUM190 cells in the presence or absence of poly(I:C) for 48h.

B. Mean fluorescence intensity of TCF-eGFP activity after poly(I:C) treatment for 6h, 24h, and 48h from 3 independent experiments. * $p < 0.05$.

Figure S6

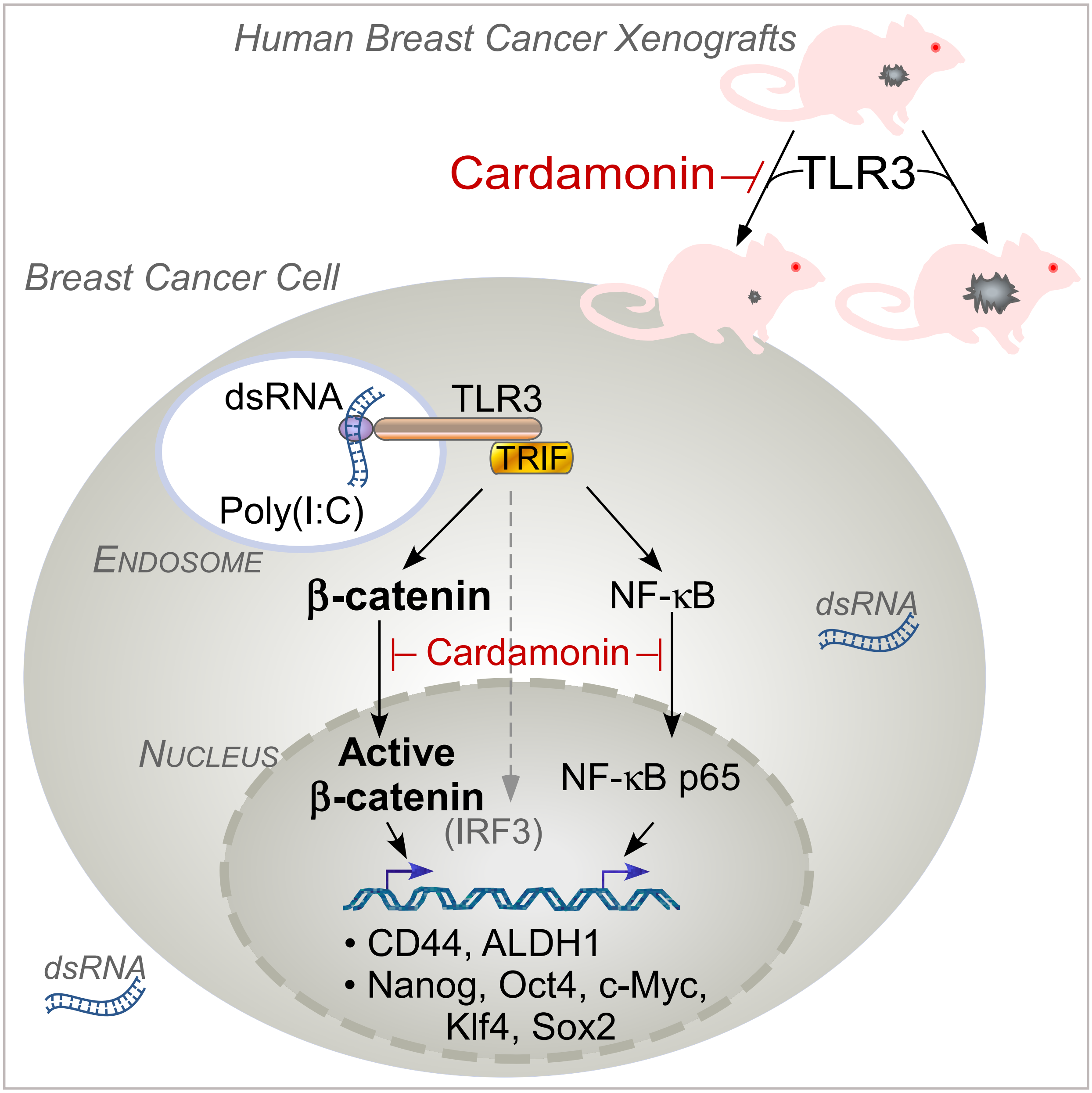

Figure S6. A graphical outline.

β-catenin pathway is required for the promotion of CSC phenotypes in breast cancer cells following TLR3 activation. Inhibition of both β-catenin and NF-κB is an effective strategy to control the growth of human breast cancer induced by TLR3 activation.
